# Changes in Young Adult Handgun Carrying in the US

**DOI:** 10.1001/jamanetworkopen.2024.58177

**Published:** 2025-02-06

**Authors:** Max A. Halvorson, Margaret R. Kuklinski, Emma Gause, Julia P. Schleimer, Heather F. Terral, Elizabeth H. Weybright, Sabrina Oesterle, Ali Rowhani-Rahbar

**Affiliations:** 1 Social Development Research Group, School of Social Work, University of Washington, Seattle; 2 Center for Climate and Health, Boston University School of Public Health, Boston, Massachusetts; 3 Firearm Injury and Policy Research Program, Department of Pediatrics, School of Medicine, University of Washington, Seattle; 4 Department of Human Development, Washington State University, Pullman; 5 Southwest Interdisciplinary Research Center, School of Social Work, Watts College of Public Service Community Solutions, Arizona State University, Phoenix; 6 Department of Epidemiology, School of Public Health, University of Washington, Seattle

## Abstract

This cohort study evaluates changes in characteristics of young aduts who carried handguns from 2019 to 2021.

## Introduction

The early 2020s were marked by an increase in firearm-related injuries^1^ and a record number of firearm-related deaths in the US.^2^ These trends coincided with events impacting US residents’ sense of safety: the COVID-19 pandemic, racial justice protests after George Floyd’s murder, and the attack on the US Capitol. Although the increase in firearm-related harm was mirrored by a rise in firearm purchases,^3,4^ it is unknown whether handgun carrying also increased.

Young adults are at high risk of interpersonal violence and can legally purchase handguns. To ensure care that promotes safety, health care and other professionals who serve young adults should understand the characteristics of young adults who carry handguns.

## Methods

Data for this cohort study are from a longitudinal study^5^ of young adults initially recruited for a community-randomized trial of the Communities That Care (CTC) prevention system, which helps communities assess and intervene on risk and protective factors related to community-specific problem behaviors in order to promote positive youth development. The sample (4407 participants) included 76% of all public school students in fifth grade in 2004 in 24 small- to moderate-sized communities across 7 states.^5^ Participants were assessed every 1 to 3 years through age 28 years. Approval was granted by the University of Washington, and written parental consent was provided at grade 5, child assent at grades 5 through 12, and youth consent after age 18 years. Reporting follows Strengthening the Reporting of Observational Studies in Epidemiology (STROBE) guidelines.

Participants reported past-year handgun carrying (“How many times in the past year [12 months] have you carried a handgun [other than while hunting or as part of your job]?”) at ages 19, 21, 23, 26, and 28 years. Gender, race, ethnicity, rurality, educational attainment, and personal income were each self-reported at age 26 or 28 years (see eMethods in Supplement 1). Race and ethnicity were assessed because to understand any potential racial and ethnic disparities in risk and protective factors, levels of outcomes, and response to the CTC intervention.

The study highlighted data at age 26 years (prepandemic, 2019) and 28 years (midpandemic, 2021) because the age 26 assessment occurred just before the events described previously and the age 28 assessment just after. Absolute and relative changes in handgun carrying from 2019 (age 26 years) to 2021 (age 28 years) were examined.

At age 28 years, we also examined whether first-time handgun carriers differed sociodemographically from continuing handgun carriers. First-time handgun carriers were those who reported handgun carrying only at 28 years. Continuing carriers were those who reported carrying a handgun at age 28 years and at least 1 time point prior (19, 21, 23, or 26 years). We assessed whether handgun carrying differed in intervention vs control communities. No differences were found; therefore, we report on the full sample. *P *values were calculated using 2-sided tests and significance was set to <.05. R version 4.4.2 (R Project for Statistical Computing) was used for analyses, which were completed from July 2023 to November 2024.

## Results

The analysis sample (3347 participants; 1806 [54.1%] cisgender female; 615 [18.4%] Hispanic, 181 [5.4%] American Indian, and 2555 [76.4%] White) consisted of 1483 participants (44.3%) who lived in cities with populations under 50 000 (Table 1). Handgun carrying prevalence increased from 11.0% (368) in 2019 to 12.9% (432) in 2021 (χ^2^_1_ = 13.0; *P* < .001).

**Table 1.** Sample Description and Comparison of Handgun Carrying at Ages 26 (2019) and 28 Years (2021) by Sociodemographic Characteristic

Characteristic	Individuals, No. (%)	Any past-year handgun carrying^a^				
		No. (%)		*P* value for χ^2^ test^b^	Absolute % increase	Relative % increase
		Age 26	Age 28			
Gender						
Cisgender female	1806 (54.1)	94 (5.2)	127 (7.0)	.003	1.8	34.6
Cisgender male	1489 (44.6)	273 (18.3)	302 (20.3)	.04	2.0	10.9
Transgender female/woman	4 (0.1)	NA^c^	NA	NA^c^	NA	NA
Transgender male/man	6 (0.2)	NA	NA	NA	NA	NA
Gender nonconforming	26 (0.8)	NA	NA	NA	NA	NA
Race and ethnicity						
Asian or Asian American	62 (1.9)	5 (8.1)	8 (12.9)	.37	4.8	59.3
Black or African American	116 (3.5)	8 (6.9)	11 (9.5)	.55	2.6	37.7
Hispanic	615 (18.4)	54 (8.8)	73 (11.9)	.01	3.1	35.2
Middle Eastern or North African	9 (0.3)	NA	NA	NA	NA	NA
American Indian or Alaska Native	181 (5.4)	24 (13.3)	30 (16.6)	.18	3.3	24.8
Native Hawaiian or other Pacific Islander	21 (0.6)	NA	5 (23.8)	NA	NA	NA
White, non-Hispanic	2555 (76.4)	295 (11.5)	332 (13.0)	.02	1.5	13.0
Multiracial	116 (3.5)	14 (12.1)	18 (15.5)	.39	3.4	28.1
Other^d^	248 (7.4)	21 (8.5)	30 (12.1)	.08	3.6	42.4
Rural vs urban						
Farm or country	341 (10.2)	64 (18.8)	66 (19.4)	.88	0.6	3.2
Small city (<50 000)	1483 (44.3)	161 (10.9)	194 (13.1)	.006	2.2	20.2
Medium city (50 000-100 000)	846 (25.3)	89 (10.5)	109 (12.9)	.03	2.4	22.9
Urban (>100 000)	675 (20.2)	54 (8.0)	63 (9.3)	.31	1.3	16.3
Educational attainment						
Less than HS	262 (7.8)	37 (14.1)	37 (14.1)	>.99	0.0	0.0
HS diploma	502 (15.0)	55 (11.0)	85 (16.9)	<.001	5.9	53.6
Some secondary	1707 (51.1)	212 (12.4)	244 (14.3)	.02	1.9	15.3
BA or higher	868 (26.0)	63 (7.3)	64 (7.4)	>.99	0.1	1.4
Personal income, US $						
<30 000	1838 (55.1)	148 (8.1)	176 (9.6)	.03	1.5	18.5
30 000-59 999	1184 (35.5)	155 (13.1)	187 (15.8)	.005	2.7	20.6
60 000-89 999	243 (7.3)	50 (20.6)	53 (21.8)	.71	1.2	5.8
≥90 000	68 (2.0)	14 (20.6)	15 (22.1)	>.99	1.5	7.3

Abbreviations: BA, Bachelor of Arts; HS, high school; NA, not available.

^a^ Analysis sample is 3347 participants who answered the handgun carrying item at both age 26 and age 28 years.

^b^ McNemar χ^2^ tests compared the likelihood of handgun carrying at age 26 and age 28 years, accounting for correlation between repeated measures.

^c^ Results for categories with fewer than 5 people endorsing handgun carrying at either time point were not reported due to concerns about low power and instability (indicated by NA).

^d^ Other race or ethnicity indicates that participants selected an “other” checkbox.

Handgun carrying increased from 2019 to 2021 within each sociodemographic group studied. Absolute increases ranged up to 5.9% and relative increases ranged up to 59.3% (Table 1). Relative increases were larger for groups with lower prevalence of handgun carrying in 2019. Compared with continuing carriers, new carriers were more likely to be women and to report annual income under $30 000 (Table 2).

**Table 2.** Comparison of Sociodemographic Characteristics of New Handgun Carriers and Continuing Handgun Carriers at Age 28 (2021)

Characteristic	Participants, No. (%)		*P* value for X^2^ test^c^
	Continuing carriers (n = 308)^a^	New carriers (n = 146)^b^	
Gender			
Cisgender female	65 (22.0)	62 (45.9)	<.001
Cisgender male	230 (77.7)	72 (53.3)	
Transgender female/woman	NA^d^	NA	
Transgender male/man	NA	NA	
Gender nonconforming	NA	NA	
Race and ethnicity^e^			
Asian or Asian American	7 (2.3)	NA	NA
Black or African American	8 (2.6)	NA	NA
Hispanic	48 (15.6)	29 (19.9)	.32
Middle Eastern or North African	NA	NA	NA
American Indian or Alaska Native	25 (8.1)	7 (4.8)	.27
Native Hawaiian or other Pacific Islander	NA	NA	NA
White, non-Hispanic	229 (77.4)	103 (75.7)	.80
Multiracial	17 (5.5)	NA	NA
Other^f^	21 (6.8)	9 (6.2)	.95
Rural vs urban			
Farm or country	24 (8.2)	10 (7.4)	.97
Small city (<50 000)	47 (16.1)	20 (14.8)	
Medium city (50 000-100 000)	128 (43.8)	60 (44.4)	
Urban (>100 000)	93 (31.8)	45 (33.3)	
Educational attainment			
Less than HS	25 (8.5)	12 (8.9)	.28
HS diploma	51 (17.3)	34 (25.2)	
Some secondary	173 (58.6)	71 (52.6)	
BA or higher	46 (15.6)	18 (13.3)	
Personal income, US $			
<30 000	100 (33.9)	76 (55.5)	<.001
30 000-59 999	135 (45.8)	53 (38.7)	
60 000-89 999	47 (15.9)	6 (4.4)	
≥90 000	13 (4.4)	NA	

Abbreviations: BA, Bachelor of Arts; HS, high school; NA, not available.

^a^ Continuing carriers were those who had carried handguns at age 19, 21, 23, or 26 years in addition to at age 28 years.

^b^ New carriers were those who carried handguns for the first time at age 28 years in 2021.

^c^ χ^2^ analyses compared whether proportions of each sociodemographic variable were identical for new carriers and continuing handgun carriers.

^d^ Results for categories with fewer than 5 people endorsing handgun carrying in either group were not reported due to concerns about low power and instability (indicated by NA).

^e^ Racial and ethnic groups were tested individually (eg, Asian vs non-Asian) because groups were not mutually exclusive.

^f^ Other race or ethnicity indicates that participants selected an “other” checkbox.

## Discussion

This study documented a rise in young adult handgun carrying in a community longitudinal study from 2019 (prepandemic) to 2021 (midpandemic). Increases in past-year handgun carrying were larger for groups with historically lower prevalence of firearm ownership, and women and individuals with low income were more likely to be first-time handgun carriers in 2021. Study limitations included a nonnationally representative sample and relatively small sample sizes for racial and gender minorities. Best practices for minimizing firearm-related injuries should be tailored to the diverse individuals who carry firearms.

## Notes

Supplement 1.
eMethods.

eReferences.

Supplement 2.
Data Sharing Statement

## References

[1] Bliton JN, Paul J, Smith AD, . Increases in adolescent firearm injuries were associated with school closures during COVID-19. Injury. 2023;54(8):110824. doi:10.1016/j.injury.2023.05.055 37296010 PMC10246889

[2] Gramlich J. What the data says about gun deaths in the U.S. Pew Research Center. 2023. Accessed November 2, 2023. https://www.pewresearch.org/short-reads/2023/04/26/what-the-data-says-about-gun-deaths-in-the-u-s/

[3] Lyons VH, Haviland MJ, Azrael D, . Firearm purchasing and storage during the COVID-19 pandemic. Inj Prev. 2021;27(1):87-92. doi:10.1136/injuryprev-2020-043872 32943492

[4] Miller M, Zhang W, Azrael D. Firearm purchasing during the COVID-19 pandemic: results from the 2021 National Firearms Survey. Ann Intern Med. 2022;175(2):219-225. doi:10.7326/M21-3423 34928699 PMC8697522

[5] Hawkins JD, Brown EC, Oesterle S, Arthur MW, Abbott RD, Catalano RF. Early effects of Communities That Care on targeted risks and initiation of delinquent behavior and substance use. J Adolesc Health. 2008;43(1):15-22. doi:10.1016/j.jadohealth.2008.01.022 18565433 PMC3867289

