# Supplementary material for: Changes in Young Adult Handgun Carrying in the US
Source: JAMA Netw Open. 2025 Feb 6;8(2):e2458177. doi: 10.1001/jamanetworkopen.2024.58177 (PMC11803477; doi:10.1001/jamanetworkopen.2024.58177)
Supplement: Supplement 1. — eMethods. eReferences. [file jamanetwopen-e2458177-s001.pdf]

Supplemental Online Content

Halvorson MA, Kuklinski MR, Gause E, et al. Changes in young adult handgun carrying from 2019 to 2021 in a longitudinal community cohort. *JAMA Netw Open*. 2025;8(2):e2458177. doi:10.1001/jamanetworkopen.2024.58177

eMethods.

eReferences.

This supplemental material has been provided by the authors to give readers additional information about their work.

© 2024 Halvorson MA et al. *JAMA Network Open*.

eMethods

Participants

Communities were matched on sociodemographic variables and randomly assigned to the intervention or control condition. Intervention activities took place when students were in grades 6 through 9. The funding agency was not involved in the collection, analysis, and interpretation of the data, or in the decision to approve publication of the finished manuscript. The sample was repeatedly surveyed across twelve waves between grade 5 (age 11, 2004) and age 28 (2021). The current study primarily used data collected at ages 26 and 28, but also drew upon data collected when the panel was 19, 21, and 23 years old to define handgun carrying history prior to age 26. Additional information on the study is available in previously published work.[1,2]

Participants responded primarily via online surveys (92% at age 26, 98% at age 28); the remainder filled out a paper-and-pencil survey. Completion incentives were $50 at ages 26 and 28. Ninety participants were deceased or no longer active in the study by age 28, bringing the active sample to 4317. Retention of the still-living sample was 88% at age 26 and 82% at age 28, and the analysis sample consisted of 3347 participants who provided data on handgun carrying at both age 26 (n = 3759) and age 28 (n = 3518). Participants who provided handgun carrying data at only one time point were not any more or less likely to carry handguns at age 26 or 28 than those who provided two data points, but were more likely to be male, black, Hispanic, Native, or other race, from large cities, and have attained less than a bachelor's degree.

Measures

© 2024 Halvorson MA et al. *JAMA Network Open*.

Data were collected using the Youth Development Survey.[20] Reporting follows the STROBE reporting guidelines.

Response options (Never, 1-2 occasions, 3-5, 6-9, 10-19, 20-29, 30-39, 40 or more) for **handgun carrying** were dichotomized such that 0 indicated no past-year handgun carrying, and 1 indicated any past-year handgun carrying.

Participant **gender** was reported at age 26 using the following response options: “Male/Man,” “Female/Woman,” “Trans Male/Trans Man,” “Trans Female/Trans Woman,” “Gender Queer/Gender Non-Conforming,” and “Something Else.” **Race** was reported at age 28 using the response options listed in Table 1. Participants reported their **ethnicity** through a separate item at age 28 as Hispanic or non-Hispanic. In order to be inclusive within each racial or ethnic group, we created dummy codes for each category. For example, an Asian participant who also identified as Hispanic would be coded as 1 on both Asian and Hispanic dummy codes. For consistency with prior work, we created a non-Hispanic White category from responses to race and ethnicity items. Detailed information on sociodemographic characteristics is available in Table 1. Missing race and ethnicity data were filled in using data from earlier time points. The "Other" category reflects participants who selected an "Other" box presented to participants. The "Multiracial" category reflects participants who selected more than one of the race categories presented. Race and ethnicity were assessed in order to understand any potential racial and ethnic disparities in risk and protective factors, levels of outcomes, and response to the CTC intervention. Race and ethnicity have also been included as covariates in models of CTC's impact to enhance the precision of the intervention effect. **Rurality** was reported at age 26 using the item “Which of the following best describes

© 2024 Halvorson MA et al. *JAMA Network Open*.

the type of area where you lived or stayed most of the time in the past 12 months?” with six response options that were collapsed to four categories: “Farm or country,” “Small city (50,000 or fewer residents),” “Medium city (50,000-100,000),” and “Urban (100,000 or more).” **Educational attainment** at age 26 was assessed by asking participants for their highest degree completed (e.g., "Associate's degree [AA/AS]") and was coded for analyses into four categories: "Less than high school," "High school diploma," "Some secondary" (which included some college, vocational school, and associate's degrees), and "Bachelor's degree or higher." **Personal income** at age 26 was self-reported in income bands and coded into four levels for analyses: $0-$29,999, $30,000-$59,999, $60,000-$89,999, and $90,000 and above.

Analysis Plan

For groups that were extremely underrepresented in the sample (e.g., gender minority participants reporting handgun carrying), we reported frequencies, but refrained from performing statistical tests. All analyses were initially conducted separately in the intervention and control samples. Sample demographics and findings did not differ by intervention condition; thus, we present results in the full sample.

eReferences

1. Kuklinski MR, Oesterle S, Briney JS, Hawkins JD. Long-term impacts and benefit-cost analysis of the Communities That Care prevention system at age 23, 12 years after baseline. *Prev Sci*. 2021;22(4):452-463. doi:10.1007/s11121-021-01218-7

© 2024 Halvorson MA et al. *JAMA Network Open*.

2. Oesterle S, Kuklinski MR, Hawkins JD, Skinner ML, Guttmannova K, Rhew IC. Long-term effects of the Communities That Care trial on substance use, antisocial behavior, and violence through age 21 years. *Am J Public Health*. 2018;108(5):659-665. doi:10.2105/AJPH.2018.304320

© 2024 Halvorson MA et al. *JAMA Network Open*.
