# Supplementary material for: Changes in Young Adult Handgun Carrying in the US
Source: JAMA Netw Open. 2025 Feb 6;8(2):e2458177. doi: 10.1001/jamanetworkopen.2024.58177 (PMC11803477; doi:10.1001/jamanetworkopen.2024.58177)
Supplement: Supplement 2. — Data Sharing Statement [file jamanetwopen-e2458177-s002.pdf]

Data Sharing Statement

Halvorson. Changes in Young Adult Handgun Carrying in the US. *JAMA Netw Open*. Published February 06, 2025. doi:10.1001/jamanetworkopen.2024.58177

Data
Data available: No

Additional Information
Explanation for why data not available: The data, analytic code, and materials necessary to reproduce the analyses are available from the first author upon reasonable request. Data were not made publicly available in accordance with informed consent from participants and in order to safeguard participants' privacy and confidentiality.
